# Obesity in Early Life: Its Causes, Prevention and Risks in Later Life

**DOI:** 10.3390/nu15132999

**Published:** 2023-06-30

**Authors:** Pieter Sauer

**Affiliations:** Department of Pediatrics, Beatrix Children’s Hospital, University Medical Center, Hanzeplein 1, 9700RB Groningen, The Netherlands; saupie46@gmail.com

Being overweight or obese at the end of the first year of life is associated with an increased risk of obesity in later life and an increased risk of developing other diseases, like type 1 diabetes mellitus, cardiovascular disorders and psycho-social problems [1]. Therefore, it is essential to avoid becoming overweight in the first year of life. Well-known risk factors for becoming overweight in the first year of life include a high BMI of the mother before pregnancy, high weight gain of the mother during pregnancy, smoking by the mother, a low level of physical activity of both the mother and the infant, nutrition during the first year of life, a high or low birth weight of the infant, a high level of weight gain during the first year of life, and the gut microbiome [1,2,3]. In a study in the Netherlands, we found that the risk of becoming overweight in young infants increases when children are brought by car to pre-school instead of walking or biking to school [3]. An environment that is stressful for infants might also increase the risk of developing overweight and obesity [4], as shown in the study by Wood et al. in this Special Issue.

In this Special Issue, Wood et al. showed that positive affect from caregivers might reduce the incidence of overweight and obesity [4]. They found that positive affect, when given by the mother or father, reduced the risk for overweight and obesity. Positive affect given by other caregivers had little effect. Thus, not only the attention given to an infant but also the social environment in which it occurs is important.

Diseases related to obesity are most likely not related to the overweight per se but to the high-fat content of an infant. Body fat content seems much more important than weight. A high weight does not necessarily indicate a high fat content, and an average weight does not mean a low fat content [5]. In a group of Hispanic adolescents with an increased BMI, we found that increased levels of insulin, plasma leptin and Hs-CRP were only present in adolescents with a high BMI and a high fat content, but not in adolescents with a high BMI and standard fat content [5]. Musalek et al. showed in their contribution to this special issue a lower physical fitness and more inadequate foodhabits in children with a normal BMI but high fat mass [6]. It might be more critical to reduce the amount of body fat than weight reduction to prevent diseases like diabetes and cardiovascular diseases.

Another factor that might affect the development of overweight is environmental pollutants. Several studies reviewed by Berghuis et al. [7] reported the adverse effects of persistent environmental pollutants on the growth and development of newborn infants. These studies showed lower birthweight and lower thyroid levels in highly exposed infants. However, whether lower birth weight and lower thyroid levels are related to the development of overweight and obesity is unclear. In this Special Issue, Berghuis et al. showed that higher levels of the contaminants, PCB, OH-PCB and PBDEs, in mothers were associated with metabolic cardiovascular risk markers for developing obesity and cardiovascular diseases in their offspring when measured during adolescence, especially in boys [8].

Overweight is also related to the type of food consumed by infants. Infants seem to prefer unhealthy food products, like hamburgers and sweetened drinks, and do not like healthy products, such as vegetables and fruits. Their dislike of healthy foods might be related to a lack of exposure to these food items. In this Special Issue, a large-scale study by Hanraths et al. examined if repeated vegetable and fruit exposure at a primary school [9] might improve children’s intake of these food items and result in eating less of the so-called unhealthy food items. This study showed that repeated exposure to vegetables and fruits had limited effects on children’s familiarity and intake of these food items [9]. More studies are needed on how to increase the intake of fruits and vegetables in infants and children. A study involving Indigenous children in Canada showed that repeated tasting sessions along with the inclusion of the whole family might be needed to change food habits. The delivery of fruits and vegetables with recipes for preparing these foods positively affected the intake of fruits and vegetables [10].

One highly debated topic in the development of overweight and adiposity in infants is the role of breastfeeding. Some studies have shown a lower risk of developing overweight in breastfed infants [11], while this is not found in other studies and reviews [12,13]. Most studies have indicated that breastfeeding must be given for at least 4–6 months to show a difference between breast- and bottle-fed infants [9]. There are a number of explanations for the conflicting findings.

First, for ethical reasons, it is impossible to conduct a randomized controlled study on the potential effect of breastfeeding. At the same time, mothers who are willing to breastfeed might be different from mothers who prefer bottle feeding. Secondly, studies can only compare the risk of overweight among breast- and bottle-fed infants or between infants receiving different formulae. A higher risk of developing obesity in bottle-fed infants might be related to the composition of the formula used, and not to the positive effect of breastfeeding. Different nutritional components, which are different between breast milk and formulae, might be responsible for the higher risk of overweight/obesity in formula-fed infants. Macronutrient components that have been studied in more detail are the protein content and the fatty acid composition of formulae, the gut flora, and differences between fore- and hindmilk.

Essential fatty acids, which are the derivatives of the parent compounds linoleic (*n*-6) and linolenic (*n*-3) fatty acids, have many effects on our human metabolism. Studies using cell cultures and animal studies have shown that these derivatives, especially arachidonic acid (AA), eicosapentaenoic acid (EPA) and docosahexaenoic acid (DHA), influence lipogenesis and might, therefore, influence the development of overweight and obesity in children. In this Special Issue, Demmelmaier and Koletzko [14] showed that, in contrast to data obtained from animal studies, studies conducted in humans do not show convincing evidence for an effect of these essential fatty acids present in human milk on the development of BMI or fat mass in infants. More studies on the potential effects of these fatty acids are needed.

A highly debated and studied factor that might explain the higher risk for overweight and obesity in formula-fed infants is the protein content of infant formulae. Rolland-Cachera was the first to show a relationship between protein intake and the incidence of overweight in infants [15]. The protein content of breast milk decreases with the duration of lactation from approximately 2.0 g/100 kcal soon after birth to 1.3 g/100 kcal at 6 months and 1.2 g at 12 months [16]. Regular infant formulae given during the first months of life have a protein content of approximately 2.0 g/100 kcal. This level does not decrease with increasing infant age. Two important studies found that infants who were fed a formula with a higher protein content had a higher weight compared to breastfed infants, but the higher weight was due to a higher protein content, rather than a higher fat mass [17,18]. Prentice et al. showed that the % protein in human milk is positively related to BMI at 12 months, while the total caloric intake of human milk is associated with a lower BMI at 12 months [19]. The study conducted by Koletzko et al. is a well-known study on the effect of a lower protein content in formulae given during the first months of life. The authors showed that infants who were fed a formula with a protein level of 1.8 g instead of 2.9 g of protein/100 kcal had a lower BMI compared to breastfed infants at two years of age [20]. In a follow-up study, the prevalence of obesity at six years of age was not different between a low-protein group and a breastfed comparison group [21]. It is still to be determined what the optimal protein content of formula feeding is. A number of studies discussed by van Kouwenhoven et al. in this Special Issue [22] found some conflicting results. One study indicated that a formula providing 1.4 g of protein/100 kcal yielded adequate growth. However, weight gain was less than infants receiving a formula containing 1.9 g or 2.2 g of protein/100 kcal during the first four months of life [23].

Another difference between breast and bottle feeding is the difference between fore- and hindmilk, with the composition of the formula being constant during formula feeding. Hindmilk is richer in fat and energy than foremilk. The protein content of hindmilk is higher than foremilk, while foremilk contains a higher level of free amino acids [24]. Karatas et al. [25] found a lower level of ghrelin and an increased level of triglycerides and leptin in hindmilk compared to foremilk. These differences might be associated with the satiety feeling at the end of breastfeeding, which is not present after formula feeding.

Based on their own studies and a review of the literature, van Kouwenhoven et al. conclude in their paper in this Special Issue that the protein level of formula feeding should mimic the decrease in protein content of human milk with increasing postnatal age and be less than 1.6 g/100 kcal [22]. Additionally, more studies are needed regarding the optimal amino acid composition of the formula used.

When comparing the risk of developing overweight and obesity in breast- and formula-fed infants, it is essential that studies provide the exact composition of the formula used in the study. When formulae were first introduced more than 50 years ago, they contained a rather high protein content. These formulae were introduced shortly after the discovery of essential amino acids, and the higher protein content was chosen to prevent insufficient essential amino acid intake. Secondly, there were fears of low absorption of animal protein. The higher risk of formula-fed infants compared to human milk-fed infants to develop overweight or obesity in the first years of life might, therefore, be related to the higher protein intake of infants fed older formulae and not to a particular component in human milk.

The risk of developing overweight is also related to the mode of delivery and the mother’s microbiome. Children born by cesarean section are more likely to develop overweight/obesity [26]. The microbiome of an obese mother is different from that of a mother without obesity. This microbiome is transmitted to the infant, which might result in overweight and obesity in the offspring [26].

All recommendations on optimal feeding for young infants to reduce the risk of developing overweight and adiposity are based on studies in groups of infants. These recommendations are not adapted to individual cases. In their contribution to this Special Issue, Baranowski et al. investigated precision pediatric nutrition adjusted to individual infants’ needs [27]. In other words, what is good nutrition for one infant might have adverse effects on other infants. For instance, the advice for an infant born with a high birth weight must differ from that for an infant with intra-uterine growth retardation. Also, adaptations might be needed according to an infant’s growth pattern. In their paper, Baranowski et al. advise how to individualize the nutritional intake of each individual child [27].

In conclusion, many prenatal and early postnatal factors influence the risk of a newborn infant for developing overweight and obesity in the first years of life. Nutrition is one of the essential factors, and it is a factor that can be influenced. The mother’s nutrition before and during pregnancy is vital, as well as the infant’s nutrition during the first year of life. Breastfeeding should be recommended, but the risk of developing overweight might not be higher when a modern formula with a lower protein content is given. The feeding of a newborn infant must be adjusted to the infant’s unique needs.
